# Influence of age on radiomic features in ^18^F-FDG PET in normal breast tissue and in breast cancer tumors

**DOI:** 10.18632/oncotarget.25762

**Published:** 2018-07-20

**Authors:** Sarah Boughdad, Christophe Nioche, Fanny Orlhac, Laurine Jehl, Laurence Champion, Irène Buvat

**Affiliations:** ^1^ IMIV, CEA, Inserm, CNRS, Université Paris-Sud, Université Paris-Saclay, CEA-SHFJ, Orsay, France; ^2^ Nuclear Medicine Department, Institut Curie, Site St-Cloud, France

**Keywords:** breast tissue, breast cancer, ^18^F-FDG PET/CT, radiomic features, influence of age

## Abstract

**Background:**

To help interpret measurements in breast tissue and breast tumors from ^18^F-FDG PET scans, we studied the influence of age in measurements of PET parameters in normal breast tissue and in a breast cancer (BC) population.

**Results:**

522 women were included: 331 pts without history of BC (B-VOI) and 191 patients with BC (T-VOI). In B-VOI, there were significant differences between all age groups for Standardized Uptake Values (SUVs) and for 12 textural indices (TI) whereas histogram-based indices (HBI) did not vary between age groups. SUV values decreased over time whereas Homogeneity increased. We had a total of 210 T-VOI and no significant differences were found according to the histological type between 190 ductal carcinoma and 18 lobular carcinoma. Conversely, according to BC subtype most differences in PET parameters between age groups were found in Triple-Negative tumors (52) for 9 TI. On post-hoc Hochberg, most differences were found between the <45 year old (PRE) group and POST groups in NBT and in Triple-Negative tumors.

**Conclusion:**

We found significant SUVs and TI differences as a function of age in normal breast tissue and in BC radiomic phenotype with Triple-Negative tumors being the most affected. Our findings suggest that age should be taken into account as a co-covariable in radiomic models.

**Methods:**

Patients were classified in 3 age groups: <45 yo (PRE), ≥45 and <55 yo (PERI) and ≥55 and <85 yo (POST) and we compared PET parameters using Anova test with post-hoc Bonferroni/Hochberg analyses: SUV (max, mean and peak), HBI and TI in both breasts and in breast tumor regions.

## INTRODUCTION

Breast cancer (BC) is the most frequent cancer in western countries representing ~25% of cancer in women and remains a leading cause of death by cancer estimated at ~15% of all deaths by cancer in 2012 [1, 2]. ^18^F-fluorodeoxyglucose positron emission tomography/computed tomography (^18^F-FDG PET/CT) is currently widely used in locally advanced breast cancer for initial staging, tumor response assessment or for detection of recurrence [3, 4, 5, 6]. Previous studies have shown the prognostic value of PET radiomic features (RF) especially SUVmax (maximum standardized uptake value) and metabolic tumor volume (MTV) in breast cancer [7, 8]. Breast cancer is a very heterogeneous disease and current RF such as SUVmax and MTV do not reflect tumor heterogeneity [9]. Radiomics is a rapidly expanding scientific field and consists in extracting image features that might reflect tumor heterogeneity, such as texture indices (TI), and in determining their relationship to histological, molecular or even genetic patterns of the lesion of interest and patient response to therapy and survival [10, 11]. Accounting for TI for the management of BC patients could be facilitated by the knowledge of the values taken by these indices in the non-pathological mammary gland. We hypothesize that TI extracted from normal or pathological breast tissue in PET may vary according to physiological changes in women's life [12, 13, 14, 15], possibly introducing a confounding factor in their interpretation. Indeed, although it is known in the literature that significant changes occur in breast tissue function and architecture throughout various periods of women's life, the influence of age in RF variations has not been explored in ^18^F-FDG PET/CT. In addition to the best of our knowledge, the age variable has never been accounted for in radiomic models [13, 16], although its usefulness has already been suggested.

The aim of our study was therefore to characterize the variations of SUV, histogram-based and TI values in ^18^F-FDG PET/CT in non-pathological breast tissue (NBT) and in a cohort of BC patients as a function of age for two purposes: 1) demonstrate the influence of age on texture analysis in normal breast tissue in comparison to other healthy soft tissues; 2) study the effect of age on BC tumors, according to the histological type and BC molecular subtype. Our findings should help determine whether age should be used as a covariate in future radiomic models especially in BC patients.

## RESULTS

### Patients

**Figure 1:**
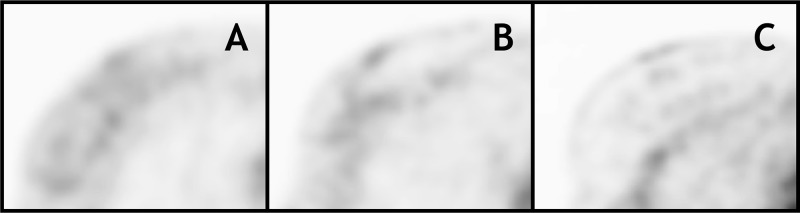
^18^F-FDG PET axial slices of the right breast in patients with non pathological breast (**A**) PRE group: 36 yo patient, (**B**) PERI group: 53 yo and (C) POST group: 66 yo patient.

A total of 326 NBT patients free of BC and other gynecological cancers were included with 652 B-VOI drawn in both breasts with a minimum volume of 14 mL. Information on hormone replacement therapy (HRT) or a history of oophorectomy was available for 168 patients yielding the exclusion of 14 and 3 patients respectively. As a result, 309 patients and 618 B-VOI were included (Figure 1, Table 1A). There were significant differences in age in our 3 age groups (<45 yo (PRE), ≥45 and <55 yo (PERI) and ≥55 and <85 yo (POST)) when using ordinary one way Anova and Hochberg and Bonferroni tests on post-hoc analysis (Table 1B).

**Table 1A:** Patient characteristics in NBT group for VOI repartition according to age group in breast, muscle and fat tissues

		PRE	PERI	POST
NBT patients	B-VOI	144	118	356
	M-VOI	39	32	125
	F-VOI	59	68	185

**Table 1B:** SUVs, TLG, HBI and TI mean and SD values according to each age group in breast tissue of NBT subjects

	PRE	PERI	POST
SUVmean^**^	0.85 ± 0.03	0.7 ± 0.03	0.55 ± 0.02
SUVmax^**^	1.6 ± 0.04	1.4 ± 0.04	1.2 ± 0.03
SUVpeak^**^	1.3 ± 0.03	1.2 ± 0.04	0.9 ± 0.03
SkewnessH^**^	0.21 ± 0.06	0.21 ± 0.07	0.48 ± 0.05
KurtosisH^*^	2.9 ± 0.11	2.8 ± 0.13	3.2 ± 0.09
EntropyH	1.4 ± 0.03	1.4 ± 0.04	1.5 ± 0.03
EnergyH	0.07 ± 0.01	0.07 ± 0.01	0.06 ± 0.01
Homogeneity^**^	0.81 ± 0.01	0.83 ± 0.01	0.86 ± 0.00
Entropy^**^	0.9 ± 0.02	0.85 ± 0.02	0.7 ± 0.02
SRE^**^	0.54 ± 0.01	0.50 ± 0.01	0.43 ± 0.01
LRE^**^	7.6 ± 0.4	8.3 ± 0.5	11.3 ± 0.3
LGZE^**^	0.31 ± 0.01	0.33 ± 0.01	0.38 ± 0.01
HGZE^*^	12.8 ± 0.6	10.5 ± 0.6	7.5 ± 0.4

^*^p value < 0.05 and ^**^p value < 0.01 on Anova test.

Among the NBT subjects, we were able to draw a region in the gluteus muscle (M-VOI) with a volume greater than 14 mL in 196 patients and region in the gluteus subcutaneous fat (F-VOI) in 312 patients (Table 1A).

Variation according to age in breast tissue of NBT subjects

There were no significant differences in SUVmean, SUVmax, SUVpeak, HBI and TI between left and right breast tissue (Mann-Withney tests, *p* > 0.05) which allowed us to pool all B-VOI together for subsequent analysis.

**Figure 2:**
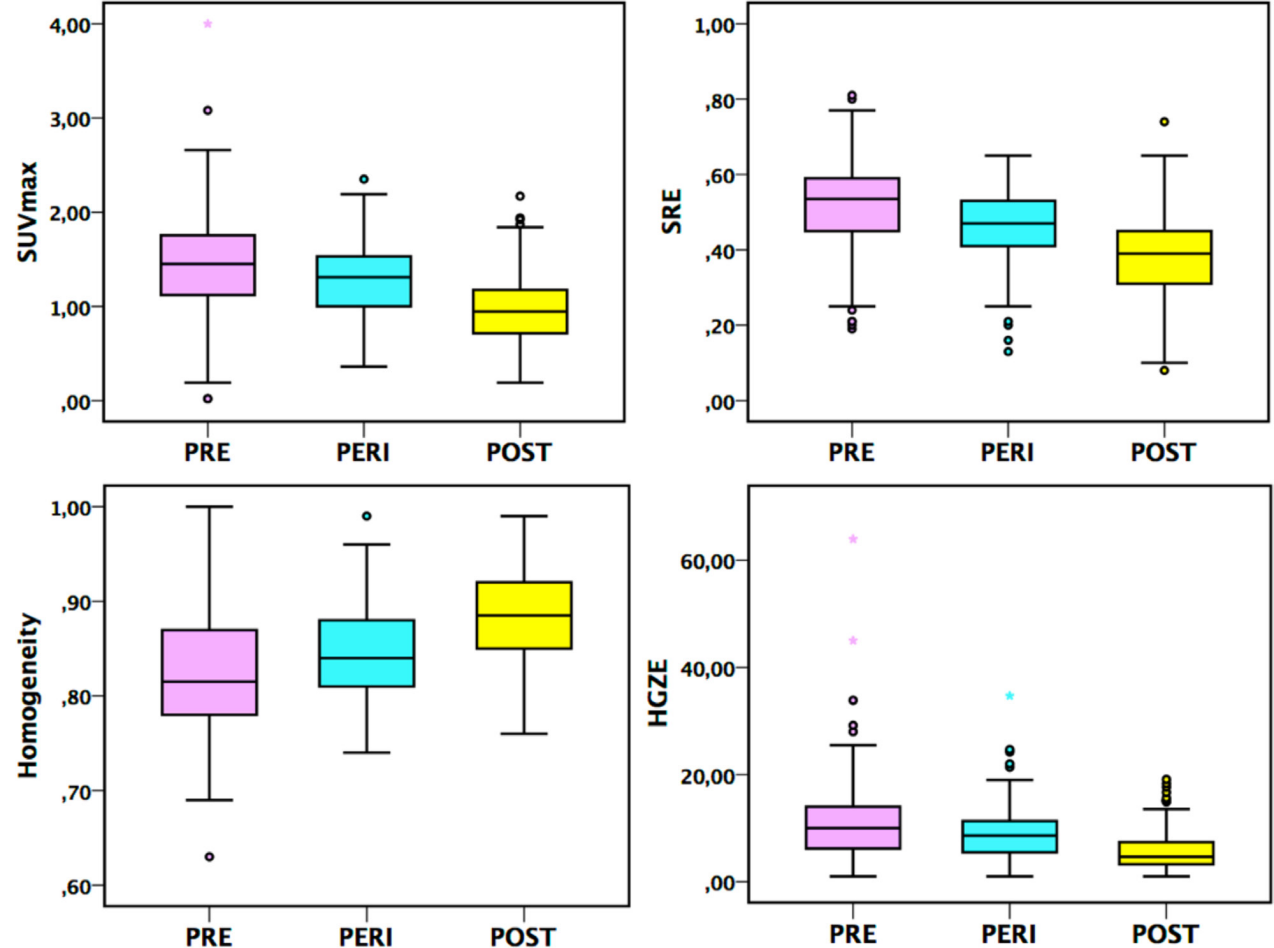
Box-plots for SUVmax and 3 TI (Homogeneity, SRE and HGZE) in the PRE, PERI and POST groups, *p* < 0.05 on Hochberg test between each age group in breast tissue of NBT subjects

Similarly, there were significant differences in SUVmean, SUVmax, SUVpeak, 2 HBI (SkewnessH and KurtosisH) and 28 TI (including the 6 robust TI) between the age groups according to Anova test (*p* < 0.05, Table 1, Figure 2, Supplementary Table 2). On post-hoc analyses, there were significant differences between all age groups for SUVmean, SUVmax, SUVpeak and 12 TI including Homogeneity, Short-Run Emphasis (SRE) and High Gray-level Zone Emphasis (HGZE) (*p* < 0.05, Figure 2, Supplementary Table 3). For other TI, including Entropy, Long-Run Emphasis (LRE) and Low Gray-level Zone Emphasis (LGZE) most differences were found between PRE and POST groups and PERI and POST groups except for GLNU and ZP for which significant differences were found only between PRE and both PERI and POST groups. For SkewnessH, KurtosisH and EnergyH differences were found between PERI and POST groups and also between PRE and POST groups for the former. SUV values decreased over time whereas for TI, the trends varied as a function of the index (Table 2).

**Table 2:** Evolution of Mean and SD values for SUVs, HBI and TI between PRE and PERI groups

		PRE/PERI	PRE/POST	PERI/POST
Breast tissue (NBT)	SUVmean	↘↘	↘↘	↘↘
	SUVmax	↘↘	↘↘	↘↘
	SUVpeak	↘↘	↘↘	↘↘
	SkewnessH	-	↗↗	↗↗
	KurtosisH	-	-	↗
	EntropyH	-	-	-
	EnergyH	-	-	-
	Homogeneity	↗	↗↗	↗↗
	Entropy	-	↘↘	↘↘
	SRE	↘↘	↘↘	↘↘
	LRE	-	↗↗	↗↗
	LGZE	-	↗↗	↗↗
	HGZE	↘↘	↘↘	↘↘
Muscle tissue (NBT)	SUVmean	-	↗	-
	SUVmax	-	↗	-
	SUVpeak	-	↗↗	-
	SkewnessH	-	-	-
	KurtosisH	-	-	-
	EntropyH	-	-	-
	EnergyH	-	-	-
	Homogeneity	↘	↘↘	-
	Entropy	↗	↗↗	-
	SRE	↗	↗	-
	LRE	↗	↗	-
	LGZE	-	↘	-
	HGZE	-	↗	-
Fat tissue (NBT)	SUVmean	-	↗	-
	SUVmax	-	↗	-
	SUVpeak	-	↗	-
	SkewnessH	-	-	-
	KurtosisH	-	-	-
	EntropyH	-	↗	-
	EnergyH	-	↘	-
	Homogeneity	-	-	-
	Entropy	-	-	-
	SRE	-	↘↘	-
	LRE	-	↗	-
	LGZE	-	↘	-
	HGZE	-	-	-

PRE/PERI, PRE and POST groups: PRE/POST and PERI and POST groups: PERI/POST. Tukey's test: ↘, ↘↘ and ↗, ↗↗ correspond to a decrease or an increase with *p* value respectively < 0.05 and < 0.01 and - no significant difference. Results are given for B-VOI, M-VOI and F-VOI.

**Figure 3:**
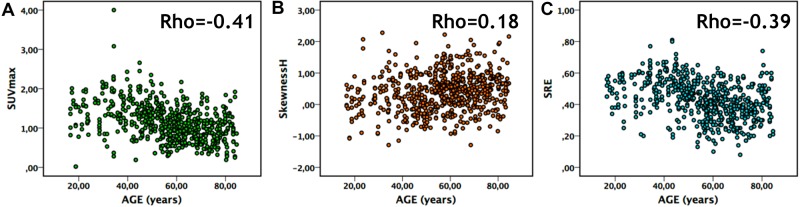
Correlation in normal breast tissue between: (**A**) age and SUVmax, (**B**) age and SkewnessH and (**C**) age and SRE.

Looking at all B-VOI (618) there were moderate (0.3<|R|≤0.5) correlations between age and SUVmean, SUVmax, SUVpeak and 18 TI in B-VOI including Homogeneity, Entropy, SRE, LRE and HGZE (*p* < 0.05, Table 3 and Figure 3, Supplementary Data 3). Correlation |R| between age and HBI in breast tissue was less than 0.18.

**Table 3:** Spearman correlation coefficients between age and PET parameters in breast, muscle and fat tissues respectively; ^*^p < 0.05 and moderate correlation (0.3< |R| <0.5)

	Breast	Muscle	Fat
SUVmean	−0.42^*^	0.35^*^	0.31^*^
SUVmax	−0.41^*^	0.34^*^	0.24
SUVpeak	−0.42^*^	0.38^*^	0.29
SkewnessH	0.18	−0.01	−0.04
KurtosisH	0.10	0.03	−0.02
EntropyH	−0.05	0.02	0.12
EnergyH	0.04	−0.08	−0.12
Homogeneity	0.36^*^	−0.25	0.14
Entropy	−0.34^*^	0.27	−0.12
SRE	−0.39^*^	0.22	−0.18
LRE	0.38^*^	−0.25	0.20
LGZE	0.29	−0.28	−0.21
HGZE	−0.39^*^	0.31^*^	0.21

### Comparison to other healthy soft tissues in NBT subjects

In muscle tissue, there were significant differences between the 3 age groups PRE, PERI and POST on Anova tests for SUVs, TLG and 16 TI including Homogeneity, Entropy, SRE, LRE, LGZE and HGZE (*p* < 0.05, Table 2, Supplementary Table 3). On post-hoc Bonferroni/Hochberg tests, unlike in breast tissue most differences were found between PRE and PERI groups and PRE and POST groups but none were found between PERI and POST groups (Table 2, Supplementary Table 3). We showed an increase of SUVs over time in muscle. TI such as Homogeneity decreased in muscle tissue between younger (PRE) and older patients (PERI and POST) without significant variations between PERI and POST groups (Table 2, Supplementary Table 3), while Entropy had the opposite trend (increase between PRE and PERI/POST). There were no significant variations for HBI between age groups except for EntropyH and EnergyH between PRE and POST groups (Supplementary Table 3).

Compared to breast tissue, there were fewer moderate correlations found between age and PET parameters in muscle, and they were observed for all SUVs and for 5 TI and HGZE alone for the main TI (Table 3, Supplementary Table 4).

In fat tissue, there were significant differences on Anova tests for SUVs, 2 HBI (EntropyH and EnergyH) and 9 TI including SRE, LRE and LGZE. On post-hoc Bonferroni/Hochberg tests, differences were only found between PRE and POST groups for SUVs, 2 HBI and 9 TI (*p* < 0.05). For SRLGE, differences were also found between PERI and POST groups. There were very few variations in SUVs, HBI or TI and there were only found between PRE and POST groups (Table 2, Supplementary Table 3).

In fat tissue, there was no substantial correlation between age and PET parameters except for SUVmean (|R|<0.3) (Table 3, Supplementary Table 4).

### Influence of age in BC tumors

In 191 BC patients with a total of 210 lesions (5 patients had bilateral BC and 14 had at least bifocal lesions), we had 190 invasive ductal carcinoma (IDC) tumors, 18 invasive ductal carcinoma (ILC) tumors and 2 tumors with mixed histological types. The most frequent BC subtype was Luminal B HER2− (Lum B HER2−) with 90 tumors. For the other BC subtype expressing HR, we had 33 and 35 tumors for Luminal A (Lum A) and Luminal B HER2+ (Lum B HER2+) subtypes respectively. There were 52 Triple-Negative (TN) tumors which did not express HR nor HER2.

**Figure 4:**
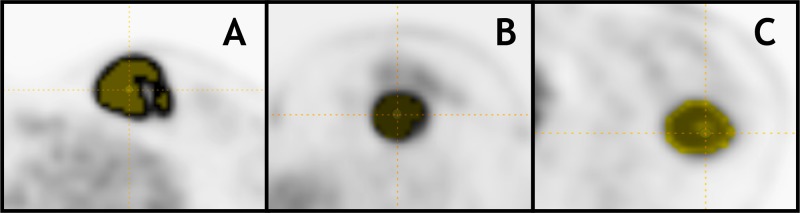
^18^F-FDG PET axial slices with 40% of SUVmax T-VOI of Triple-Negative tumors with BC stage T2N0M0 in patients from different age groups: (**A**) 33yo patient (PRE): SUVmax = 28.1, Homogeneity = 0.47, SRE = 0.87 and LGZE = 0.000, (**B**) 47yo patient (PERI): SUVmax = 11.9, Homogeneity = 0.28, SRE = 0.96 and LGZE = 0.002 and (**C**) 77yo patient (POST): SUVmax = 6.8, Homogeneity = 0.41 SRE = 0.92 and LGZE = 0.007.

When looking at the differences in PET parameters according to the 3 age groups previously defined in BC patients in the all cohort we did not found any significant differences between age groups on Anova test and post-hoc analysis. There were some weak correlations between age and SUVpeak and 10 TI including Homogeneity, SRE and LRE (Supplementary Tables 5 and 6). Looking at the influence of age according to the histological type there were no significant differences between age groups in 190 IDC and 18 ILC tumors or significant correlations on Spearman test. When focusing on each BC subtype, most differences in PET parameters between age groups found on Anova test were in 52 TN tumors for 9 TI including Homogeneity, Entropy, SRE and LGZE (*p* < 0.05) and on post-hoc analysis those differences were found between PRE and POST groups (Figure 4, Table 4 and Supplementary Table 5). Similarly, we found significant weak to moderate associations in TN tumors between age and SUVmean, SUVmax and 18 TI including Homogeneity, SRE, LRE, LGZE and HGZE (*p* < 0.05, Table 5 and Supplementary Table 6). For the other BC subtypes, including Lum B HER2− subtype which had the biggest population with a total of 90 tumors there were almost no dependency between radiomic features and age (Supplementary Tables 5 and 6). HER2 positive (HER2+) tumors were excluded because of their very low number (12 tumors not included in our BC cohort).

**Table 4:** SUVs, TLG, HBI and TI mean and SD values according to each age group in BC patients with TN tumors

	PRE (28 T-VOI)	PERI (12 T-VOI)	POST (12 T-VOI)
SUVmean	8.4 ± 3.6	6.7 ± 3.9	6.2 ± 0.02
SUVmax	13.7 ± 5.6	10.8 ± 5.8	10 ± 7.3
SUVpeak	11.7 ± 4.9	8.9 ± 5.4	8.6 ± 6.2
MTV	10.4 ± 7.7	6.4 ± 4.5	11.1 ± 9.7
TLG	89.6 ± 77	53.7 ± 77.8	66.5 ± 73.5
SkewnessH	0.49 ± 0.32	0.59 ± 0.45	0.44 ± 0.31
KurtosisH	2.5 ± 0.5	2.7 ± 0.8	2.4 ± 0.4
EntropyH	1.7 ± 0.1	1.6 ± 0.1	1.7 ± 0.1
EnergyH	0.02 ± 0.00	0.03 ± 0.01	0.03 ± 0.01
Homogeneity^*^	0.29 ± 0.08	0.34 ± 0.1	0.39 ± 0.2
Entropy^*^	2.2 ± 0.3	2.1 ± 0.4	1.9 ± 0.5
SRE^*^	0.95 ± 0.03	0.94 ± 0.04	0.91 ± 0.08
LRE	1.3 ± 0.4	1.3 ± 0.3	1.6 ± 0.7
LGZE^*^	0.00 ± 0.00	0.01 ± 0.01	0.02 ± 0.02
HGZE	808.5 ± 610.4	582.9 ± 594.4	553.4 ± 678.2

^*^p value < 0.05 and ^**^p value < 0.01 on Anova test.

**Table 5:** Spearman correlation coefficients between age and PET parameters in Lum A, Lum B HER2−, Lum B HER2+ and TN tumors respectively; (^*^p < 0.05) and ^**^p < 0.1

	Lum A	Lum B HER2−	Lum B HER2+	TN
Number of tumors	33	90	35	52
SUVmean	−0.31^**^	0.13	−0.22	−0.29^*^
SUVmax	−0.27	0.13	−0.24	−0.29^*^
SUVpeak	−0.30	0.09	−0.20	−0.25
SkewnessH	0.18	−0.05	−0.08	−0.04
KurtosisH	0.27	−0.04	0.03	0.03
EntropyH	−0.20	0.03	0.04	0.00
EnergyH	0.25	−0.04	−0.03	−0.07
Homogeneity	0.26	−0.11	0.25	0.28^*^
Entropy	−0.30^**^	0.08	−0.21	−0.19
SRE	−0.27	0.08	−0.23	−0.30^*^
LRE	0.27	−0.08	0.21	0.29^*^
LGZE	0.27	−0.14	0.24	0.30^*^
HGZE	−0.26	0.13	−0.24	−0.29^*^

As a significant association between age and RF was observed in TN tumors, we performed multivariate analysis without and with age as a covariate to determine whether radiomic features were significantly different as a function of Ki-67 expression, tumor grade, the presence of tumor necrosis, and the presence of CIS. We found additional significant TI when age was used as a covariate (5 TI compared to 2 TI without age as a covariate) to distinguish between low and highly proliferative TN tumors based on Ki-67 expression using 30% as a cutoff. Similarly, using age as a covariate, there was one more TI that significantly differed between TN tumors with and without necrosis. Likewise, SUVmean and 1 more TI were significantly different between TN tumors with and without CIS when age was used a covariate. By contrast, results were identical without and with accounting for age when assessing TI as a function of the tumor grade, with 23 TI being significantly different between grade II and grade III TN tumors (Supplementary Table 7).

## DISCUSSION

To the best of our knowledge, we demonstrated for the first-time significant differences in SUVs, HBI and TI in normal breast tissue according to age in ^18^F-FDG PET images. In our study, we drew a spherical B-VOI in the upper outer quadrant of the mammary gland because it has been established that it contains more lobular units [17]. We divided our population in 3 age groups depending on menopause average occurrence in our population of French women [18, 19]. We demonstrated statistically significant differences for SUVmean, SUVmax, SUVpeak, 2 HBI (SkewnessH and KurtosisH) and 28 TI between the age groups predominantly between POST and the other age groups PRE and PERI. Significant variations of PET parameters as a function of age in NBT might be explained by previous histopathological studies which demonstrated that changes occur in the breast tissue with age leading to a variation in the amounts of epithelial and connective tissues [20]. Indeed, the maximum amount in all the breast quadrants reaches a peak in the third decade followed by a rapid decline until the sixth decade suggesting that the evolution of breast tissue starts well before the onset of menopause [20]. Indeed, with age there is a scarcity of acini and lobules which also decrease in size whereas the proportion of epithelial tissue in postmenopausal women remains relatively stable [17, 20]. As the age of menopause in patients of NBT group was not recorded, some patients in the peri-menopausal/early-menopausal group could present with a sufficient estrogenic impregnation to maintain a glandular mammary gland close to the one present in childbearing age patients whereas some might present with early menopause [20–22]. This might explain smaller differences found between « PRE and PERI » groups.

To investigate the specificity of our results in NBT, we assessed if there was an influence of age on PET parameters variations in other healthy soft tissues such as muscle and fat tissues. We found few moderate correlations between age and SUVs and some TI for muscle and only with SUVmean for fat tissue, but they were all less than those observed in NBT (Table 3). In muscle tissue, most differences were found between PRE and PERI groups and PRE and POST groups, but very few between PERI and POST groups contrary to breast tissue. This promotes that changes related to age in muscle tissue occurs earlier than in breast. By contrast, in fat tissue, very few significant differences in radiomic features were found between age groups. Altogether, these results suggest that the influence of age on radiomic features extracted from ^18^F-FDG PET is less pronounced in fat and muscle than in breast tissue.

### Influence of age in BC tumors

It has been well established that age is a risk factor in BC with an increased incidence and a slower but continuous slope of increase after menopause is reached [23]. For instance the proportion of intra-lobular carcinoma increases with age specifically among women above 50 yo which may be related to postmenopausal status with an increased incidence rate reported since the late 1970s [24, 25]. In our study, we did not see significant differences in PET parameters between the 3 age groups previously defined when looking at the whole BC cohort but there were weak though significant correlations between age and various TI including Homogeneity, SRE and LRE. When studying the influence of age in IDC and ILC tumors, with the former histological type representing the vast majority of the tumors (91.3%), we did not find significant differences between age groups or correlation with age. Yet, when we looked into the influence of age according to each BC molecular subtype we found significant differences mostly in TN tumors. Conversely, the influence of age on variation of RF was almost inexistent in the other subtypes Lum A, Lum B with or without HER2 expression in agreement with the heterogenous biology of BC and hinting additional etiological differences between BC subtypes.

It has previously been reported that BC risk factors vary according to molecular subtypes which might be associated with additional etiologic heterogeneity in BC [26]. Young premenopausal women have different distribution of BC molecular subtypes compared to the general population with a higher proportion of Triple-Negative tumors [27]. Yet, it is not clear how much of the “biology” of this subtype is affected by age though it is possible that some etiological differences exist according to the age of diagnosis [27]. Nonetheless, this in agreement with our findings as 52% of TN tumors were found in BC patients <45 yo (PRE) and they had tumors with significant differences with the other age groups, including lower Homogeneity and higher Entropy and SRE than postmenopausal women (Figure 4, Table 4). Other risk factors according to the BC subtype have been reported in the literature, for instance significant association between increased body mass index and reduced risk for Lum A tumors in premenopausal women but not in TN tumors also suggesting etiological differences in their development [26]. Moreover, variations in some molecular subtypes prevalence has been shown to vary according to ethnicity [28]. Indeed, African American women tend to have a higher rate of Triple-Negative tumors compared to postmenopausal African American women and non-African women of any age which leads to a poorer prognosis of BC in the former [28]. In this context it is possible that some other factors influence BC etiology and that the significant correlations between PET parameters and age in TN tumors could be etiologically relevant especially since differences were found between pre (PRE) and postmenopausal women (POST). Triple-Negative tumors tend to be poorly differentiated and express higher levels of proliferative markers that are reflected by differences in the PET imaging phenotype [16]. In that setting, RF might bring additional information on the intrinsic heterogeneity of this subtype which presented with most statistically significant correlations between age and RF [26, 27, 29]. Depending on the age of the patient at diagnosis different etiological factors might be involved and have different prognostic implications. In that context, implementing age as a covariate in radiomic models when assessing TN tumors on ^18^F-FDG PET/CT could account for some of the heterogeneity in imaging this molecular subtype and help improving their robustness. We hypothesized that this could bring additional and independent predictive value as a prognostic factor though it deserves further investigations. As a preliminary analysis we used age as a covariate in multivariate analysis when assessing radiomic features according to some immuno-histological factors in TN tumors. This resulted in finding more significant differences in RF according to Ki-67 expression, the presence of tumor necrosis or *in situ* carcinoma in TN tumors (Supplementary Table 7). This confirms that age might account for some heterogeneity in TN tumors and should thus be used as a covariate.

The limited number of Lum B HER2+ tumors might explain the very few significant results found for this subtype.

### Comparison to literature

Some recent studies focused on pathological breast tissue and did find promising results in texture analysis in BC but did not use age as a covariate [9, 30]. The influence of age on breast tissue imaging phenotype has been well-established on mammography features with breast density varying as function of age, which has been acknowledged as an independent risk factor for developing breast cancer [13, 22, 31, 32]. In PET/CT literature, previous reports investigated the relationship between SUVs in normal tissues including normal breast tissue and body weight or breast density but those did not include HBI or TI measurements [33–35]. Some authors reported significantly higher ^18^F-FDG uptake in dense breasts than in non-dense breasts, however this did not influence the accuracy of ^18^F-FDG to detect breast malignancies [35, 36]. Kumar et al. also reported significant differences in SUVs as a function of breast tissue density and showed a trend of negative correlation between age and SUVs with a decrease over time [37].

Some *in vitro* studies in normal breast tissue have reported that expression of proliferation indices such as mitotic index or labelling index decreases with age [38–40]. Those findings might explain why there were significantly higher SUVmean, SUVmax and SUVpeak value in NBT of patients in the PRE group compared to the PERI and POST groups as it has been established that there was positive association between proliferative indices expression such as Ki-67 and ^18^F-FDG uptake including in breast cancer [41–43].

In our opinion knowing that significant variations in PET parameters as a function of age occur in breast tissue is important in the context of radiomics, where predictive models are developed from image-derived features. Our results suggest that the age variable might be important to use as a covariate in radiomic analysis of breast tumor and breast tissue. We have previously shown that RF were significantly different in the non-tumor region close to the breast tumor in comparison to healthy contralateral breast tissue in patients with unilateral BC [44]. Also, a recent study in breast DCE-MRI reported that a radiomic model including intra-tumoral and peri-tumoral features measured from pretreatment DCE-MRI better predicted pathological complete response in BC patients undergoing neoadjuvant chemotherapy than a model including radiomic features measured in the tumor only [45].

In addition, it should be underlined that harmonization of imaging protocols and image analysis is essential for institution-independent interpretation rules [10, 16, 46] of the radiomic features and for a wide use of predictive radiomic models [47–49].

### Limitations and perspectives

The unbalanced number of B-VOI in each age group is due to the fact that ^18^F-FDG PET/CT scans are most often performed in older patients who also more often present with BC [23]. Yet, our findings were confirmed using bootstrap analysis to compare PET parameters between the 3 age groups (PRE, PERI and POST) in balanced conditions thus removing the potential confounding effect introduced by different group sizes (data not shown).

In our study, the actual age of menopause was not known and the information on postmenopausal hormone replacement therapy was only available for 168 patients with a history in 8.3% of them. In addition, a history of oophorectomy was found in 1.8% of the patients. In France as in most western countries, the latest recommendations restrict the prescription of substitutive treatment to menopausal women with important menopausal symptoms so troublesome as to affect quality of life and to a maximum of 5 years [50]. This is consistent with the small proportion of patients with a history of HRT (8.3%) among those for whom this information was available. The stage of the cycle at the time of the PET/CT was not taken into account as per usual in routine practice in most nuclear medicine departments. Nevertheless, higher expression of proliferative indices in the second half of the menstrual cycle suggests that this factor might be of interest for further investigations in NBT [38–40]. Overall, the PRE, PERI and POST age groups were defined according to epidemiological data in the source population [18, 19] and the sample size in each group is in our opinion sufficient to diminish the influence of the confounding factors previously described.

## MATERIALS AND METHODS

### Patients

Patients were recruited retrospectively in our institution from March 2010 to December 2015. Computerized patient records were used to retrieve ^18^F-FDG PET/CT referral information and to review patients clinical history when available for patients treated in our institution.

### NBT population

Patients who underwent ^18^F-FDG PET/CT at initial staging for malignant diseases (carcinoma, lymphoma predominantly) prior to any treatment were included. We excluded patients with a history of local treatment (breast surgery, radiotherapy etc.), gynecological and non-gynecological malignancies or chemotherapy in the last five years before the ^18^F-FDG PET/CT scan was performed.

All scans in which a suspicious focal ^18^F-FDG uptake was seen in breast tissue on first ^18^F-FDG PET/CT visual analysis were excluded for further analysis.

Patients were classified in 3 age groups defined given the average time of occurrence for menopause in our French women population [18, 19]: childbearing age <45-year-old (yo) (PRE); peri-menopausal/early menopausal women >45 and <55 yo (PERI) and post-menopausal women >55 and <85 yo (POST). The stage of the cycle at which the patients underwent the scan was not taken into account and is not recorded in routine practice in our institution. Information regarding the use of postmenopausal HRT was not always available but when it was, patients with history of HRT were excluded. We did not include pregnant and breast-feeding women and patients under 16 yo or over 85 yo to avoid introducing biases associated with extreme values.

### BC patient population

We included all BC patients who underwent ^18^F-FDG PET/CT at initial staging before receiving any treatment (chemotherapy, endocrine treatment or radiotherapy) with no prior history of BC and excluding patients with metastatic disease to reduce confounding factors. All BC tumors analyzed in this study were histologically proven on the core needle biopsy performed prior to treatment initiation, including in patients with multiple BC. Clinical stage was revised according to the American Joint Committee on Cancer (AJCC) 8th edition [51]. Patient data were collected anonymously from computerized patient's records consisting of AJCC staging and histopathological parameters assessed on the diagnostic core needle biopsy. The histopathological parameters of interest in this study were: histological type classified in two categories: IDC or ILC carcinoma, and the molecular subtype according to current guidelines: Lum A, Lum B HER2−, Lum B HER2+, HER2+ and TN [29, 52].

Immuno-histo-chemical (IHC) tests were performed as per our institution protocol following current guidelines on formalin-fixed, paraffin embedded tissues, using specific antibodies directed against estrogen receptor and progesterone receptor with positivity defined if at least 10% of the cells expressed either one of those receptors [52]. HER2 over-expression was considered positive when more than 30% of the cells expressed c-erbB-2 onco-protein (HER2 3+) or if FISH (fluorescence *in situ* hybridization) testing was positive for HER 2+ tumors.

### PET/CT protocol

Patients received an intravenous injection of 3–3.5 MBq/kg of ^18^F-FDG when capillary blood glucose level was below 11 mmol/L and after 6 hours fasting. Whole body imaging PET/CT scans (from the vertex to mid-thighs) were acquired from 60 to 80 minutes after ^18^F-FDG injection (Discovery 690 PET/CT scanner, GE Healthcare, Waukesha, WI, USA) at 2.5 min per bed position. A low-dose whole body CT scan without contrast medium was performed prior to the PET acquisition. Images were reconstructed using an ordered-subset expectation maximization iterative reconstruction algorithm (2 iterations, 24 subsets) and post-filtered using a 6.0 mm full width at half maximum Gaussian filter. The reconstructed PET image voxel size was 2.7 × 2.7 × 3.3 mm^3^.

### Image analysis

Images were analyzed by a senior nuclear medicine physician using the LIFEx software (www.lifexsoft.org) [53]. In NBT subjects, a spherical breast volume of interest (B-VOI) was drawn in one breast and mirrored to the contralateral breast in the external quadrants of the mammary gland avoiding the retro-areolar region to improve reproducibility.

In addition, for each NBT subject, one spherical volume of interest was drawn in the gluteus muscle (M-VOI) and one in the gluteus subcutaneous fat (F-VOI). We aimed for a B-VOI, M-VOI and F-VOI size greater than 14 mL to get meaningful values for TI and avoid biases due to the known relationship between TI and volume for small volumes [47, 54]. In BC patients, a coarse tumor volume of interest was manually drawn and we applied an automatic segmentation method consisting of a fixed threshold set to 40% of the maximum SUV, defining a tumor region called T-VOI thereafter.

Within B-VOI, M-VOI, F-VOI and T-VOI, we calculated SUV, histogram-based indices (HBI) and TI from 4 different matrices: Co-occurence, Gray-Level Run Length, Neighborhood Gray-level dependency and Gray-level zone length matrix (Supplementary Table 1). Measurements included: SUVmax (maximum SUV standardized using the body weight), SUVmean (average value in the VOI), SUVpeak (average value in a sphere of 1 mL at a position that maximizes the average value in the sphere), MTV (Metabolic Tumor Volume), TLG (equal to SUVmean x MTV), four histogram-based features (SkewnessH, KurtosisH, EntropyH and EnergyH) and thirty-one TI calculated after absolute resampling of gray-levels between 0 and 20 SUV units, with a bin width equal to 0.3 SUV [47], (Supplementary Table 1). Among the 31 TI, 6 have previously been shown to be both robust with respect to region delineation and relatively independent one from another [47] and will be called “robust” TI thereafter: Homogeneity, Entropy, LRE, SRE, LGZE and HGZE.

### Statistical analysis

In B-VOI, M-VOI and F-VOI of NBT subjects, Anova tests were used to compare SUV, HBI and TI values between the age groups. Bonferroni/Hochberg tests including correction for multiple testing were used in the post-hoc analysis to determine between which groups the significant differences were found.

Correlations were characterized using Spearman correlation coefficients between PET parameters derived from B-VOI, M-VOI, F-VOI and age.

In BC patients, we used Anova tests to compare SUV, HBI and TI values between 3 age groups previously defined in NBT group for the whole BC cohort, according to the histological type and according to the BC molecular subtype. Bonferroni/Hochberg tests including correction for multiple testing were used in the post-hoc analysis to determine between which groups the significant differences were found.

Correlations were characterized using Spearman correlation coefficients between PET parameters derived from T-VOI and age for the whole cohort, according to the histological type and to the BC subtype.

To determine the potential additional value of taking age into account when analyzing tumor heterogeneity, we implemented age as a covariate in multivariate analysis when assessing radiomic tumor heterogeneity as a function of Ki-67 expression (30% cut-off to distinguish between high and low proliferative tumors), tumor grade (grade II vs grade III), the presence of tumor necrosis, and the presence of *in situ* carcinoma (CIS). These analyses were done in BC molecular subtypes for which we observed significant associations between age and RF in T-VOI.

All tests were two-sided and *p*-values less than or equal to 0.05 were interpreted as statistically significant. Analyses were performed using IBM SPSS Statistics v22.0.

## CONCLUSIONS

We demonstrated significant SUV and TI differences in normal breast tissue as a function of age, which are most likely to be related to physiological changes occurring in the mammary gland. This age-dependency of radiomic features was greater in breast tissue than in muscle, even if few differences were also observed, and in fat tissues, where radiomic features were barely affected by age. The influence of age in BC radiomic phenotype varied according to BC molecular subtypes with Triple-Negative tumors being the most affected. Our findings suggest that age should be taken into account as a covariate in radiomic models, especially in Triple-Negative tumors.

## SUPPLEMENTARY MATERIALS TABLES













## Abbreviations

B-VOI: a spherical breast volume of interest
BC: Breast cancer
^18^F-FDG PET/CT: 18F-fluorodeoxyglucose positron emission tomography/computed tomography
F-VOI: a spherical volume of interest in fat tissue
HBI: histogram-based indices
HGZE: High Gray-level Zone Emphasis
HER2 positive: HER2+
LGZE: Low Gray-level Zone Emphasis
LRE: Long-Run Emphasis
Luminal A: Lum A
Luminal B HER2-: Lum B HER2-
Luminal B HER2+: Lum B HER2+
MTV: metabolic tumor volume
M-VOI: a spherical volume of interest in muscular tissue
PERI: peri-menopausal/early menopausal women >45 and <55 yo group
POST: post-menopausal women >55 and <85 yo group
PRE: childbearing age <45-year-old (yo) group
RF: Radiomic features
SRE: Short- Run Emphasis
SUVmax: maximum standardized uptake value
SUVmean: average SUV value in the VOI
SUVpeak: average SUV value in a sphere of 1 mL at a position that maximizes the average value in the sphere
TI: texture indices
Triple-Negative: TN
