# Supplementary material for: Influence of age on radiomic features in 18F-FDG PET in normal breast tissue and in breast cancer tumors
Source: Oncotarget. 2018 Jul 20;9(56):30855–68. doi: 10.18632/oncotarget.25762 (PMC6089391; doi:10.18632/oncotarget.25762)
Supplement: Supplementary file 1 [file oncotarget-09-30855-s001.pdf]

Influence of age on radiomic features in ^{18}F-FDG PET in normal breast tissue and in breast cancer tumors

SUPPLEMENTARY MATERIALS

Supplementary Table 1: Table extracted from Orlhac F, Soussan M, Maisonobe JA, Garcia CA, Vanderlinden B, Buvat I

List of indices calculated from texture matrices	
**Matrix**	**Index**
Cooccurrence Matrix	Homogeneity
	Energy
	Correlation
	Contrast
	Entropy
	Dissimilarity
Gray-Level Run Length Matrix	SRE
	LRE
	LGRE
	HGRE
	SRLGE
	SRHGE
	LRLGE
	LRHGE
	GLNUr
	RLNU
	RP
Neighborhood Gray-Level Different Matrix	Coarseness
	Contrast
	Busyness
Gray-Level Zone Length Matrix	SZE
	LZE
	LGZE
	HGZE
	SZLGE
	SZHGE
	LZLGE
	LZHGE
	GLNUz
	ZLNU
	ZP

SRE = short-run emphasis; LRE = long-run emphasis; LGRE = low grey-level run emphasis; HGRE = high grey-level run emphasis; SRLGE = short-run low grey-level emphasis; SRHGE = short-run high grey-level emphasis; LRLGE = long-run low grey-level emphasis; LRHGE = long-run high grey-level emphasis; GLNUr = grey-level non-uniformity for run, RLNU = run-length non-uniformity; RP = run percentage; SZE = short-zone emphasis; LZE = long-zone emphasis; LGZE = low grey-level zone emphasis; HGZE = high grey-level zone emphasis; SZLGE = short-zone low grey-level emphasis; SZHGE = short-zone high grey-level emphasis; LZLGE = long-zone low grey-level emphasis; LZHGE = long-zone high grey-level emphasis; GLNUz = grey-level non-uniformity for zone; ZLNU = zone length non-uniformity; ZP = zone percentage. Tumor texture analysis in 18F-FDG PET: relationships between texture parameters, histogram indices, standardized uptake values, metabolic volumes, and total lesion glycolysis. Journal of Nuclear Medicine. 2014; 55:414–22.

Supplementary Table 2: Mean and SD values for SUVs, HBI and TI in each age group PRE, PERI and POST for B-VOI, M-VOI and F-VOI; $^{*}p$ value < 0.05 and $^{**}p$ value < 0.01. See Supplementary_Table_2

Supplementary Table 3: Evolution of Mean and SD values for SUVs, HBI and TI between PRE and PERI groups: PRE/PERI, PRE and POST groups: PRE/POST and PERI and POST groups: PERI/POST for B-VOI, M-VOI and F-VOI; Hochberg test: ↘, ↘↘ and ↗, ↗↗ correspond to an increase or decrease with p value respectively < 0.05 and < 0.01 and - no significant difference. See Supplementary_Table_3

Supplementary Table 4: Spearman correlation coefficients between age and breast, muscle and fat tissues respectively; *$p < 0.05$ and $0.3<|R|\leq 0.5$

	Breast	Muscle	Fat
SUVmean	−0.42*	−0.35*	0.31
SUVmax	−0.41*	−0.34*	0.24
SUVpeak	−0.42*	−0.38*	0.29
SkewnessH	0.18	−0.01	−0.04
KurtosisH	0.10	0.03	−0.02
EntropyH	−0.05	0.02	0.12
EnergyH	0.04	−0.08	−0.12
Homogeneity	0.36*	−0.25	0.14
Energy	0.33*	−0.25	0.12
Contrast	−0.36*	0.24	−0.14
Correlation	−0.19	0.16	−0.02
Entropy	−0.34*	0.27	−0.12
Dissimilarity	−0.36*	0.25	−0.14
SRE	−0.39*	0.22	−0.18
LRE	0.38*	−0.25	0.20
LGRE	0.44*	−0.35*	−0.21
HGRE	−0.44*	0.35*	0.19
SRLGE	0.34*	−0.23	−0.27
SRHGE	−0.44*	0.34*	−0.06
LRLGE	0.44*	−0.39	0.17
LRHGE	−0.36*	0.08	0.28
GLNU	0.17	0.05	0.02
RLNU	−0.38*	0.20	−0.04
RP	−0.38*	0.24	−0.20
Coarseness	0.05	−0.16	0.21
Contrast_2	−0.19	0.12	0.06
Busyness	−0.01	−0.09	0.13
SZE	−0.07	0.04	−0.04
LZE	0.35*	−0.12	0.13
LGZE	0.29	−0.28	−0.21
HGZE	−0.39*	0.31*	0.21
SZLGE	−0.01	−0.11	−0.08
SZHGE	−0.20	0.16	0.01
LZLGE	0.42*	−0.26	0.11
LZHGE	−0.13	0.19	0.17
GLNU_2	−0.16	−0.10	−0.05
ZNLU	−0.15	0.02	0.04
ZP	−0.25	−0.02	−0.16

Supplementary Table 5: *p*-values of Anova tests comparing PET parameters between the 3 age groups (PRE, PERI and POST) according to BC histological type, subtype and for all BC tumors; $^{*}p < 0.05$ and $^{**}p < 0.1$. See Supplementary_Table_5

Supplementary Table 6: Spearman correlation coefficients between PET parameters and AGE according to BC histological type, subtype and for all BC tumors; $^{*}p < 0.05$. See Supplementary_Table_6

Supplementary Table 7: *p* values obtained on multivariate (MV) analysis comparing RF in TN tumors according to Ki-67 expression (≤30% vs >30%), the presence of tumor necrosis, in situ carcinoma and tumor grade grade (Grade I + II vs III); $^{*}p < 0.05$ and ** additional $p < 0.05$ when age is used as a co-variable in MV analysis. See Supplementary_Table_7
